# Regional Cerebral Perfusion and Cerebrovascular Reactivity in Elderly Controls With Subtle Cognitive Deficits

**DOI:** 10.3389/fnagi.2019.00019

**Published:** 2019-02-19

**Authors:** Merel van der Thiel, Cristelle Rodriguez, Dimitri Van De Ville, Panteleimon Giannakopoulos, Sven Haller

**Affiliations:** ^1^Institute of Bioengineering, École Polytechnique Fédérale de Lausanne, Lausanne, Switzerland; ^2^Faculté de Médecine, Université de Genève, Geneva, Switzerland; ^3^Division of Institutional Measures, Geneva University Hospitals, Geneva, Switzerland; ^4^CIRD – Centre d’Imagerie Rive Droite, Geneva, Switzerland; ^5^Department of Surgical Sciences, Division of Radiology, Uppsala University, Uppsala, Sweden

**Keywords:** arterial spin labeling, asymptomatic controls, cerebrovascular reactivity, brain perfusion, clinicoradiologic correlations, CO_2_

## Abstract

**Background:** Recent studies suggested that arterial spin labeling (ASL)-based measures of cerebral blood flow (CBF) as well as cerebral vasoreactivity to CO_2_ (CVR CO_2_) show significant alterations mainly in posterior neocortical areas both in mild cognitive impairment (MCI) and Alzheimer disease. It remains, however, unknown whether similar changes occur in at risk healthy elders without clinically overt symptoms. This longitudinal study investigated patterns of ASL perfusion and CVR CO_2_ as a function of the cognitive trajectories in asymptomatic elderly individuals.

**Methods:** Seventy-nine community-dwelling subjects (mean age: 78.7 years, 34 male) underwent three neuropsychological assessments during a subsequent 3-year period. Individuals were classified as stable-stable (SS), variable (V), or progressive-progressive (PP). Between-group comparisons were conducted for ASL CBF and transit-time delay maps and β-maps of CO_2_ response. Spearman’s rho maps assessed the correlation between ASL (respectively, CVR CO_2_ measures) and Shapes test for working memory, as well as Verbal fluency test for executive functions. Three group-with-continuous-covariate-interaction designs were implemented to investigate group-based differences on the association between neuropsychological scores and ASL or CO_2_ measures.

**Results:** Comparison of CBF maps demonstrates significantly lower perfusion in the V-group as to PP-cases predominantly in parietal regions, including the precuneus and, to a lesser degree, in temporal and frontal cortex. A stronger CVR CO_2_ response was found in the PP-group in left parietal areas compared to the V-group. V-cases showed a stronger ASL-Shape value relationship than V-group in right temporoparietal junction and superior parietal lobule. CO_2_-Shape value correlation was significantly higher in both SS and PP-groups compared to the V-group in right insular and superior perisylvian regions.

**Conclusion:** Our data indicate the presence of decreased ASL and CVR CO_2_ values mainly in parietal and fronto-temporal areas in cases with the first signs of cognitive instability (V-group). Importantly, the PP-group, at high risk for MCI transition, displays an increase of both parameters in the same areas. Clinicoradiologic correlations also indicate a clear distinction between the V-group and both PP and SS-cases. These data imply the presence of an inverted U-shape pattern of regional blood flow and CVR in old age that might predict subsequent cognitive fate.

## Introduction

Age-related changes in cerebral blood flow (CBF) have been related to increased risk for cognitive decline and Alzheimer disease (AD), implying that cerebrovascular mechanisms play a pivotal role in brain health and sustenance of cognition (Wierenga et al., 2014; Montagne et al., 2015; Popa-Wagner et al., 2015; Sierra-Marcos, 2017). For the past three decades, the nuclear medicine techniques Single Photon Emission Computed Tomography (SPECT) and Positron Emission Tomography (PET) have served as gold standards for perfusion and metabolism studies in brain aging and AD. These techniques, however, require use of radioactive tracers and are more expensive that the more recently developed perfusion-weighted MRI techniques. Among these latter, arterial spin labeling (ASL) uses magnetically labeled arterial blood water as a diffusible endogenous tracer and displays similar diagnostic ability to detect AD as fluoro-2-deoxy-D-glucose (FDG)-PET (Tosun et al., 2016). This technique revealed brain hypoperfusion mainly in bilateral parietal areas, precuneus, angular and posterior cingulate cortex in MCI and early AD, which overlaps with the patterns of hypometabolism on FDG-PET observed later in disease progression, indicating the potential to use ASL for early detection of cognitive decline (for review see Alsop et al., 2008; Chen et al., 2011; Haller et al., 2016; Fällmar et al., 2017; Riederer et al., 2018). The rare ASL studies showed a more diffuse hypoperfusion in posterior inferior and frontal aspects of the brain in at risk healthy controls (Tosun et al., 2016; de Vis et al., 2018). Higher ASL measured-CBF in medial frontal, lateral temporal, parietal cortex, insula, and basal ganglia was reported in APOE 𝜀4 carriers with the worst cognitive performances (Zlatar et al., 2016).

The capacity of brain vasculature to enhance blood flow in response to challenging conditions is found to be a key parameter in very early stages of neurodegeneration, even prior to the development of clinically overt cognitive deficits. In fact, higher ASL values in these cases may be the consequence of the brain vasculature’s efforts of adapting to a threatening cellular environment. Blood oxygenation level dependent (BOLD) functional MRI allows for assessment of this reactivity. Recent data has indicated that the cerebral vasoreactivity CO_2_ (CVR CO_2_) could detect significant dysfunctions both in MCI and AD cases. In contrast, whether or not similar changes occur in at risk healthy elders is still unknown (Cantin et al., 2011; Yezhuvath et al., 2012; Richiardi et al., 2015).

Within this study, both ASL perfusion and CVR CO_2_ patterns were explored in 79 community-dwelling elderly individuals who were cognitively preserved at inclusion and had undergone two neuropsychological assessments during a subsequent 3-year period. The data revealed distinct patterns of brain perfusion and cerebrovascular reactivity as a function of the cognitive trajectories of elderly controls.

## Materials and Methods

### Participants

The series used in our analysis is part of a population-based longitudinal study on healthy aging funded by the Swiss National Foundation of Research in Geneva. The research protocol was approved by the Ethics Committee of the University Hospitals of Geneva. All experimental procedures were carried out in accordance with the approved guidelines and with the principles of the Declaration of Helsinki. All participants were given written informed consent prior to inclusion. Participants were contacted via advertisements in local media to guarantee a community-based sample. Exclusion criteria included psychiatric or neurologic disorders, sustained head injury, history of major medical disorders (neoplasm or cardiac illness), alcohol or drug abuse, regular use of neuroleptics, antidepressants or psychostimulants and contraindications to MR imaging. To eliminate possible confounding effects of cardiovascular disease, individuals with subtle cardiovascular symptoms and a history of stroke and transient ischemic episodes were also excluded from the present study.

The final sample included 79 participants, classified as cognitively healthy controls (mean age 78.7 ± 3.5 years; 44 women) who underwent three neuropsychological evaluations (baseline 18 months and 36 months follow-up) and a MRI-T1 examination (only baseline). Clinical assessment included the Mini-Mental State Examination (MMSE, Folstein et al., 1975), the Lawton Instrumental Activities of Daily Living (IADL, Barberger-Gateau et al., 1992) and the Hospital Anxiety and Depression Scale (HAD, Zigmond and Snaith, 1983), the Consortium to Establish a Registry for Alzheimer’s Disease (CERAD) neuropsychological battery, Digit Span (Wechsler, 1955) and Corsi block (Milner, 1971) for verbal and visual working memory respectively, the Trail Making Test A and B (Reitan, 1958) for executive functioning, Digit-Symbol-Coding (Wechsler, 1977) for attention, Boston Naming (Kaplan et al., 1983) for language, Ghent Overlapping Figures (Schnider et al., 1997) for visual gnosis and RI-48 Cued Recall Test (RI-48) for episodic memory (Haller et al., 2017). Individuals meeting the DSM-IV criteria of dementia or for MCI on the basis of clinical and neuropsychological assessments, were excluded from the study (Haller et al., 2017). In order to explore correlations between the neuropsychological performances and imaging variables, and given the limited sample size, we selected two main tests that encompass visual working memory (Shapes test, Baddley et al., 1994) and executive functions (Phonemic verbal fluency Bruyer and Tuyumbu, 1980). Descriptive statistics and statistical differences between the SS, V, and PP group on age, gender and the neuropsychological scores were calculated with separate one-way ANOVA’s using IBM SPSS Statistics version 25.

### Neuropsychological Follow-Up

For follow-up measurements, which took place 18 months after inclusion, the cognitively healthy individuals underwent full neuropsychological assessment once again. Individuals who obtained stable cognitive scores over the baseline and follow-up evaluation were classified as stable controls. The progressive control group obtained a follow-up evaluation of at least 0.5 standard deviations (SD) lower than measured at baseline, on a minimum of two cognitive tests. Two neuropsychologists clinically assessed all individuals independently. The final classification was determined by a trained neuropsychologist taking into account both the results of the neuropsychological tests and overall clinical assessment (Xekardaki et al., 2015). All of the cases were assessed once again 18 months later with the same neuropsychological battery. The participants were subsequently grouped as described above (−0.5 SD in at least two cognitive tests), with comparison of the scores of the latest assessment. Stable individuals showing no changes in the second assessment were classified in the stable-stable (SS) group and progressive individuals demonstrating a further decline as progressive-progressive (PP). The variable group (V) refers to participants demonstrating a fluctuating scoring pattern, incorporating stable-progressive, progressive-stable or progressive-improved individuals. The final sample included 24 SS, 33 V, and 22 PP cases.

### ASL

#### MR Imaging

As described previously in more detail (van der Thiel et al., 2018), ASL imaging was performed on a 3T GE MR750w using a 32-channel head array coil. Perfusion images were acquired with a 3D stack-of-spiral fast spin echo sequence preceded by a Hadamard encoded Pseudo-Continuous ASL (PCASL) module with background suppression. A total label duration of 4 s. was encoded into seven sub-blocks. The label durations were 0.22, 0.26, 0.30, 0.37, 0.48, 0.68, and 1.18 s, post label delays were chosen to be 1.00, 1.22, 1.48, 1.78, 2.15, 2.62, and 3.32 s. The total scan time lasted for 4.02 min.

Images were created at all delay times. The combined delay map consists of the sum of the delay times per subject. Two CBF maps were used for subsequent analysis, the raw uncorrected flow maps and the transit time corrected flow maps adjusted for arterial transit time.

Key imaging parameters were: field of view (FOV) = 22.0 cm, slice thickness 4.0 mm, 32 slices, bandwidth ± 62.5 kHz, 4 arms with 640 points each. The PCASL images had a matrix size of 128 × 128 and a voxel size of 1.88 × 1.88 × 4.0. Images were acquired with an echo time of 10.5 ms and a repetition time of 5936 ms. Acquisition included a T1/PD weighted reference image with matched parameters for quantification. The T1/PD combination image was acquired with the same TR as the perfusion images (5936 ms) and was formed by saturation recovery sequence with a 2.0 s saturation time. This saturation time is the same as that of the ASL sequence. The T1 value for blood assumed in the CBF quantification was 1.60 s. The Image reconstruction was performed using IDL based recon code and reconstructed images were stored as DICOM images into the scanner’s database.

#### Data Preprocessing ASL

The ASL data was processed using the fMRI utility of the Brain Software Library (FSL, Version 5.0.9^1^). The combined delay image was obtained per participant and non-brain tissue was removed using the Brain Extraction Tool (BET^2^, part of FSL). The brain extracted combined delay maps were normalized to Montreal Neurological Institute (MNI) standard space using an Echo Planar Image (EPI) template from the Statistical Parametric Mapping (SPM8) toolbox, standard space using linear registration (FMRIB Linear Image Registration Tool^3^, part of FSL). The concatenated transformation matrix of the transit delay maps was then applied to the uncorrected flow maps, the transit time corrected flow maps and the transit delay maps to spatially normalize the data to the EPI template.

The normalized ASL images were smoothed with a 5 mm FWHM Gaussian kernel using a dilated 2 mm brain extracted MNI mask (FSLUtils ^4^, part of FSL). Due to the low intrinsic signal to noise level of ASL, we have decided to apply spatial smoothing in order to improve detection of group differences since spatial normalization intrinsically includes a certain degree of smoothing. Furthermore, some statistical procedures require smoothed data.

#### ASL Group Comparisons

All of the following analysis were executed for the corrected, uncorrected and delay maps separately. The cognitive groups were compared using voxel-wise permutation-based testing (Randomize^5^, part of FSL), with threshold-free cluster-enhancement correction for multiple comparisons applied (Smith and Nichols, 2009) and *p* < 0.05 considered significant. Five thousand permutations were executed per contrast. The MNI 2 mm brain extracted mask was used during randomization for masking of non-brain voxels. A triple *T*-test design was administered with age and gender as non-explanatory co-regressors (Liu et al., 2012). Age was defined as the difference in days between the date of birth and the day of scanning.

#### ASL-Based Clinicoradiologic Correlations

For each group, Spearman rho correlations were calculated between the Shapes test and the Verbal fluency test and the corrected, uncorrected and delay maps separately using MATLAB version R2017b, to assess clinicoradiologic correlations in each group.

Group differences in correlation between the neuropsychological scores on the Shapes test, and the Verbal fluency test and the ASL response were investigated with a continuous-covariate-interaction-design^6^.

To see whether the relationship between the ASL response and the neuropsychological test differed between groups, a triple *T*-test with 6 contrasts was carried out per neuropsychological test, representing all possible differences in scores between groups. The cognitive groups were once again compared using voxel-wise permutation-based testing with 5000 permutations per contrast (Randomize; see text footnote^5^, part of FSL), threshold-free cluster-enhancement correction for multiple comparisons applied (Smith and Nichols, 2009) masked with the 2 mm brain extracted mask and *p* < 0.05 considered significant.

### CVR CO_2_

#### CO_2_ Admission

The CO_2_ administration protocol is described in more details elsewhere (Richiardi et al., 2015). In short, the CO_2_ challenge consisted of 9 min of CO_2_ admission via a nasal cannula. A concentration of 7% CO_2_ mixed with synthetic air was given with the sequence 1 min OFF, 2 min ON, 2 min OFF, 2 min ON, 2 min OFF. Subjects were asked to breathe normally through the nose. During admission, EPI covering the entire brain was acquired using a 32 multi-channel coil with the following parameters: FOV = 28.5 cm, 96 × 96 matrix, voxel size of 2.9688 × 2.9688 × 3 mm^3^, echo times of 30 ms, repetition time of 3000 ms, 45 repetitions.

#### Preprocessing MRI

The CO_2_ data was processed using FSL Version 5.0.9 (Jenkinson et al., 2012). The functional sequences were realigned to the mean per sequence to correct for motion effects (Jenkinson et al., 2002) and non-brain tissue was removed using the Brain Extraction Tool (Smith, 2002). Individual structural images were skull-stripped and co-registered to a standard 2 mm MNI brain extracted by employing standard FLIRT procedure (Jenkinson et al., 2002).

The transformation matrices from the functional to the subject space were calculated using the mean functional images per subject with the epi_reg script provided by FSL (Jenkinson et al., 2002).

White matter (WM) and Cerebral Spinal Fluid (CSF) masks were calculated as follows; WM and CSF were segmented from the individual high-resolution 3D images with FMRIB’s automated segmentation tool (Zhang et al., 2001). The resulting masks were compared with *a priori* tissue mask containing an average of MRI images of 152 subjects, as provided by the MNI and hereafter binarized with a threshold of 0.6 in subject space. For both WM and CSF masks, a mean time series of the functional data within the tissue specific mask was calculated and used to filter out the WM and CSF effects, and additionally the motion correction parameters with the FSL command-line tool. Smoothing was applied to the denoised functional data with a 5 mm FWHM Gaussian kernel using the brain-extracted mask of the mean functional image. As for the ASL data, we applied spatial smoothing to reduce the effects of noise on the group analysis. In addition, to ensure an analysis pipeline as similar as possible for both the CO2 and ASL data, the application of spatial smoothing was appropriate. In this manner, comparability of the two techniques was optimized.

The denoised, smoothed functional CO_2_ data was normalized to the standard 2 mm MNI brain extracted template by usage of the FMRI Expert Analysis Tool v6.00, with the same normalization method employed as described earlier. In addition, a high pass filter of 270 s was applied to the data and pre-whitening was performed with FMRIB’s Improved Linear Model.

#### First-Level Analysis CVR CO_2_

The pre-processed functional CO_2_ sequences were used to carry out the first-level analysis. A standard first-level FSL pipeline was employed to fit the CO_2_ response to the subjects’ response. Convolution of the ON/OFF response of the CO_2_ admission was done using a simple square wave form, correcting for wash-in and wash-out effects in a straightforward and effective way (Mutch et al., 2012; Richiardi et al., 2015). The square wave form has shown to approximately model the increase and decrease of CO_2_ levels during administration (Richiardi et al., 2015).

#### CVR CO_2_ Response Group Comparisons

To investigate whether there was a difference in CVR CO_2_ response between the SS, V and PP group, an *F*-test design was applied. To further specify which groups differentiated from one another on the CO_2_ response, a triple *T*-test design was executed with a standard higher-level FSL pipeline.

#### CVR CO_2_ Response-Based Clinicoradiologic Correlations

Spearman rho correlation was computed between the Shapes test and Verbal fluency test and CO_2_ maps for each group, using MATLAB version R2017b. The relationship between the neuropsychological scores (the Shapes and the Verbal fluency tests) and the CVR CO_2_ response was investigated by a three group with continuous-covariate-interaction-design see text footnote^6^. To see whether the relationship between the CVR CO_2_ response and the neuropsychological test differed between groups, triple *T*-tests with 6 contrasts were carried out per neuropsychological test, covering all potential group-differences.

## Results

### Demographic Data and Neuropsychological Test Scores

No statistically significant group differences were found in demographic variables (age and gender). Neuropsychological scores at baseline did not differ between the three groups (see Table 1).

**Table 1:** Demographical and neuropsychological data of the included study groups of the SS, V, and PP participants.

	Stable-Stable		Variable		Progressive-Progressive		*P*-value			
	Mean	*SD*	Mean	*SD*	Mean	*SD*	Group	SS vs. V	V vs. PP	PP vs. SS
Gender (male)	11 M		13 M		10 M		*p* = 0.967, η^2^ = 0.000	*p* = 0.883	*p* = 0.900	*p* = 1.00
Age (years)	78.86	4.122	78.96	4.074	78.19	2.158	*p* = 0.728, η^2^ = 0.091	*p* = 0.994	*p* = 0.727	*p* = 0.810
Shapes	34.75	2.609	33.06	4.007	34.32	2.476	*p* = 0.128, η^2^ = 0.053	*p* = 0.134	*p* = 0.341	*p* = 0.894
Verbal fluency	18.00	4.625	17.85	4.912	17.59	4.896	*p* = 0.850, η^2^ = 0.004	*p* = 0.878	*p* = 0.997	*p* = 0.866
Trail A/B	2.53	0.814	2.71	0.982	2.72	0.668	*p* = 0.668, η^2^ = 0.011	*p* = 0.710	*p* = 0.997	*p* = 0.714
MMSE	28.58	1.283	28.39	1.784	28.36	1.002	*p* = 0.959, η^2^ = 0.001	*p* = 0.992	*p* = 0.979	*p* = 0.956
IADL	8.54	0.779	8.15	0.364	8.64	1.529	*p* = 0.128, η^2^ = 0.053	*p* = 0.276	*p* = 0.154	*p* = 0.938
HAD	5.54	3.623	7.73	4.837	8.50	5.087	*p* = 0.076, η^2^ = 0.066	*p* = 0.184	*p* = 0.813	*p* = 0.080
Digit span forward	7.96	1.732	9.03	2.243	8.23	1.974	*p* = 0.119, η^2^ = 0.054	*p* = 0.126	*p* = 0.326	*p* = 0.895
Digit span backward	5.25	1.511	5.88	1.691	4.86	1.754	*p* = 0.079, η^2^ = 0.065	*p* = 0.338	*p* = 0.073	*p* = 0.710
Corsi block forward	7.42	1.316	7.55	1.394	7.27	1.352	*p* = 0.766, η^2^ = 0.007	*p* = 0.934	*p* = 0.747	*p* = 0.932
Corsi block backward	6.83	0.963	6.42	1.251	6.41	1.260	*p* = 0.356, η^2^ = 0.027	*p* = 0.400	*p* = 0.999	*p* = 0.443
Boston naming	19.21	1.021	19.27	1.153	19.27	0.767	*p* = 0.967, η^2^ = 0.001	*p* = 0.970	*p* = 1.00	*p* = 0.975
Ghent overlapping	4.96	0.204	4.94	0.348	5.00	0.000	*p* = 0.682, η^2^ = 0.010	*p* = 0.958	*p* = 0.659	*p* = 0.842
CERAD	10.83	0.381	10.82	0.528	10.73	0.767	*p* = 0.790, η^2^ = 0.006	*p* = 0.995	*p* = 0.831	*p* = 0.803
Digit-symbol-coding	55.38	12.521	55.18	8.658	51.95	11.090	*p* = 0.466, η^2^ = 0.020	*p* = 0.997	*p* = 0.515	*p* = 0.523

*No significant group-differences were found on age, gender or any of the neuropsychological tests.*

### ASL Group Comparisons

**FIGURE 1:**
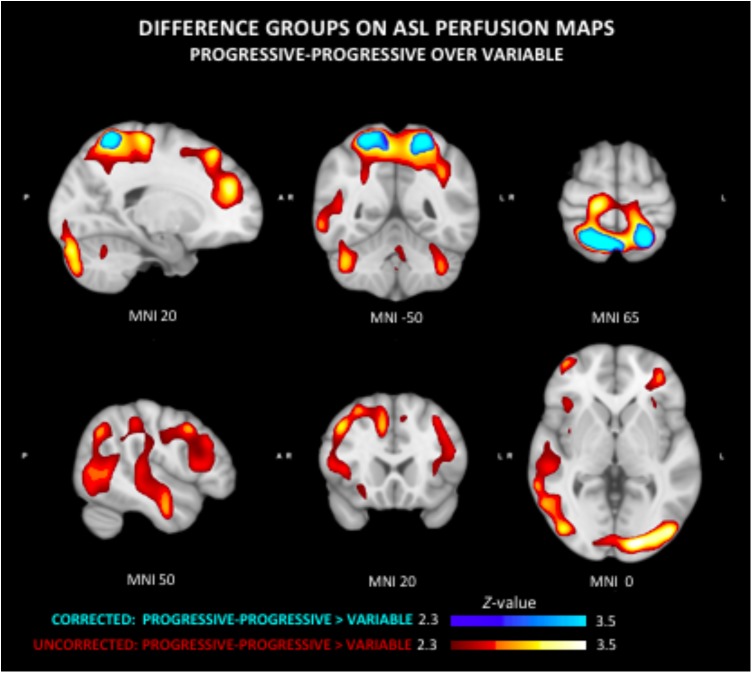
Group differences in arterial spin labeling (ASL) perfusion measures. The progressive-progressive (PP) group shows regional higher perfusion values as compared to the variable (V) group in both the uncorrected and corrected flow maps. This difference can be clearly seen in the corrected flow maps in the parietal regions including the precuneus. The PP group similarly shows significantly higher perfusion than the V group within the parietal lobe, but also temporal and frontal regions.

**FIGURE 2:**
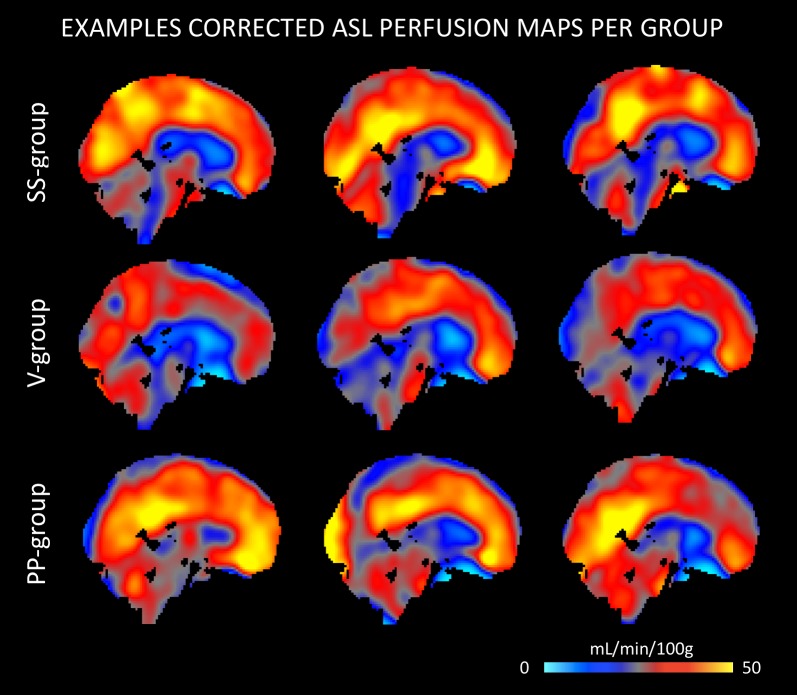
Example ASL perfusion maps of stable-stable (SS), V, and PP group separately. The examples of the V group demonstrate a lower perfusion within the precuneus, while in the example maps of both the SS and PP group perfusion in these regions remains relatively preserved.

The quantitative CBF and CVR values of the included study groups are shown in Table 2. Comparison of the perfusion maps between the three groups demonstrated a significantly higher perfusion value in both the corrected and uncorrected maps of the PP group as compared to the V group. As can be seen in Figure 1, these regions largely overlap in both perfusion maps. Differences within the corrected flow maps between the groups can be clearly seen within the parietal regions, including the precuneus but also in temporal and frontal regions. Example corrected perfusion maps from the different groups emphasize the hypoperfusion within the precuneus of the V group once more, while in the example maps of both the SS and PP group perfusion in these regions remains preserved (Figure 2).

**Table 2:** Quantitative CBF and CVR values of the included study groups of the SS, V, and PP participants.

	Stable-stable		Variable		Progressive-progressive	
	Mean	*SD*	Mean	*SD*	Mean	*SD*
**ASL CBF maps**	36.180118	16.936111	34.896856	15.598446	34.043468	15.517011
**ASL Delay maps**	1535.553350	408.553332	1535.864498	410.159331	1523.346373	426.743474
**CO_2_ CVR maps**	9.490050	25.768619	9.131709	25.240867	9.982506	25.039040

Group comparison of the uncorrected maps demonstrates a significantly higher perfusion in PP compared to the V group that was diffusely present in several neocortical areas. No other significant group differences were found in respect to ASL delay maps.

### ASL-Based Clinicoradiologic Correlations

**FIGURE 3:**
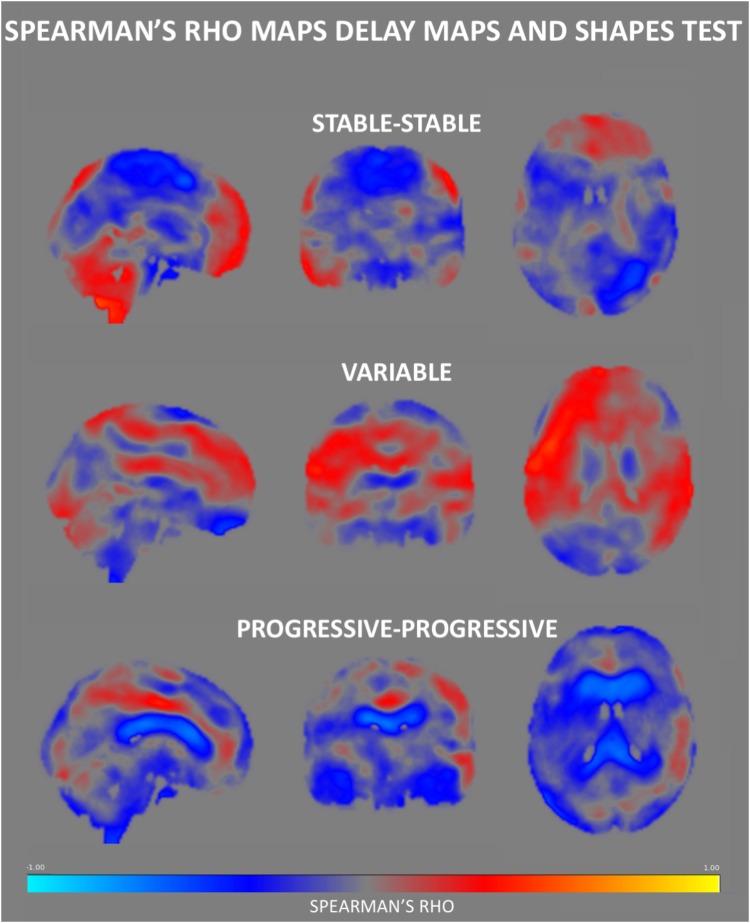
Spearman’s rho correlation maps between ASL values and the Shapes test performance. A predominance of negative associations was found in the SS group, with positive correlations residing in the frontal and cerebellar regions. The PP group also displays a negative association in most of the brain areas especially in regions surrounding the ventricles. Interestingly, the Spearman rho maps of the V group show global prominent positive correlations in areas that show negative rho values in the SS group, residing largely in the medial part of the brain.

**FIGURE 4:**
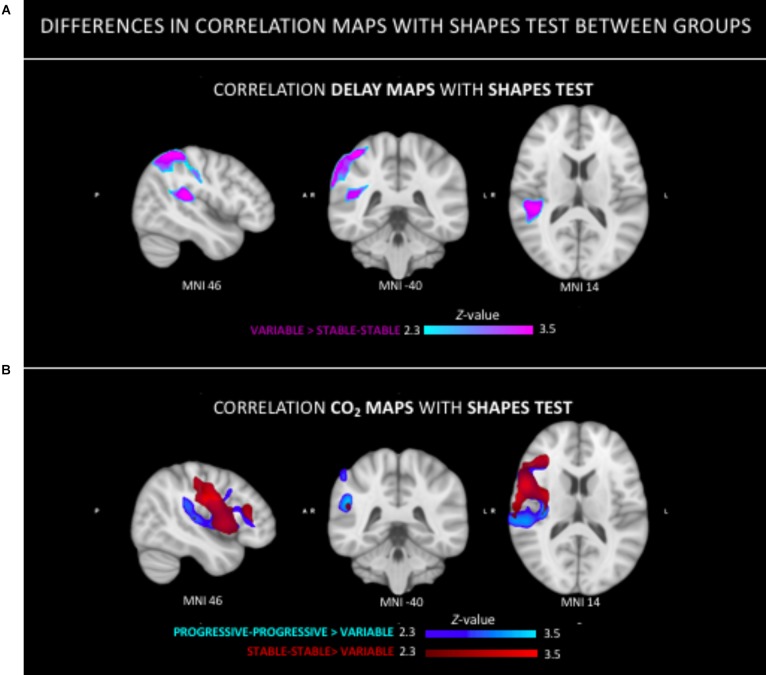
Group differences in the relation between neuroimaging parameters (ASL, CVR-CO_2_) and Shapes test performance. **(A)** Stronger positive relationship in the tempo-parietal junction and superior parietal lobe in V group as compared to the SS group. **(B)** Stronger positive correlation in both the PP and SS group as compared to the V group in right peri-sylvian and superior areas.

The Spearman correlation maps per group reveal distinct patterns as a function of the group of reference only for the Shapes test. In SS group, negative associations between ASL values and Shapes test performances were found in most areas with the exception of the frontal and cerebellar regions (Figure 3). A similar predominance of negative correlations was observed in PP group. Interestingly, the Spearman rho maps of the V group show prominent positive correlations in areas that show negative rho values in the SS group. Group comparisons showed significant differences in ASL-Shapes test correlation between the V and SS group in right temporo-parietal junction and superior parietal lobule (Figure 4A).

### CVR CO_2_ Response Group Comparisons

**FIGURE 5:**
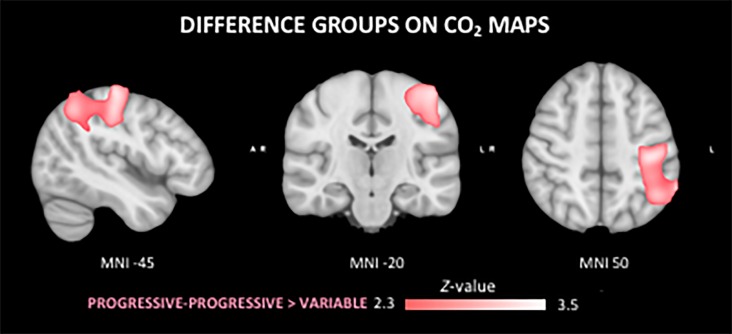
Differences in the cerebral vasoreactivity to CO_2_ (CVR CO_2_) response between groups. The PP group shows a stronger CVR CO_2_ response in comparison to the V group in the superior left parietal lobe.

The *F*-test demonstrated that there were significant differences between groups on the CO_2_ response (data not shown). The *T*-tests demonstrated a significantly stronger CO_2_ response of the PP group in left parietal areas as compared to the V group (Figure 5). No regional differences in CO_2_ response were found between the other groups.

### CVR CO_2_ Response-Based Clinicoradiologic Correlations

**FIGURE 6:**
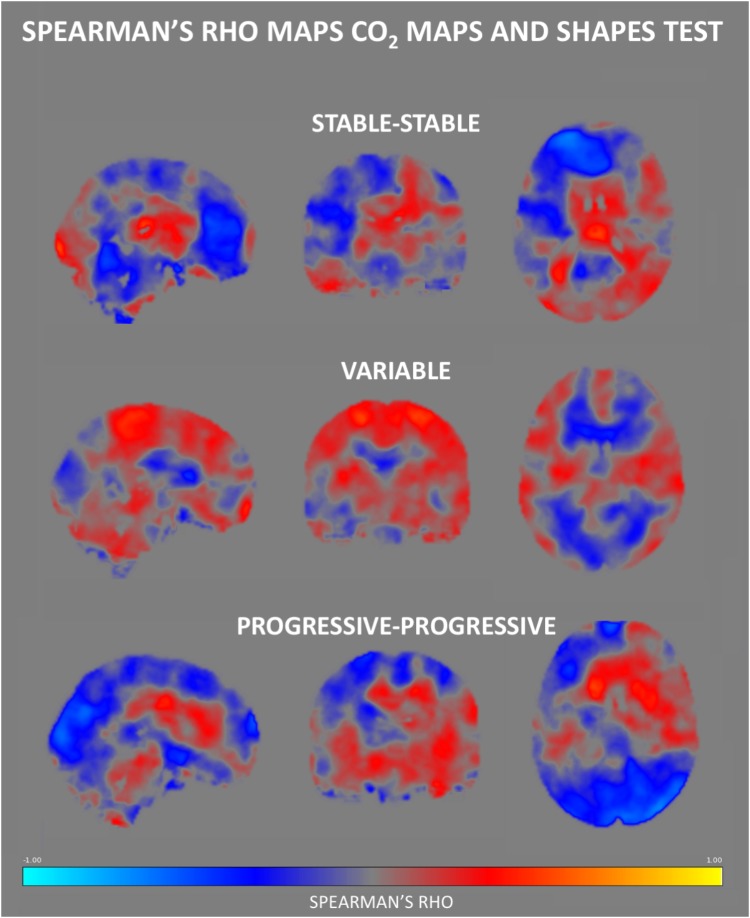
Regional Spearman’s rho correlation maps between CVR CO_2_ response and the Shapes test performance. Both the SS and PP groups displayed a fairly similar pattern of correlation, showing a positive relationship in the medial brain, with a frontal display of negative rho values. In contrast, the V group demonstrates negative correlations in medial brain regions, along with positive ones in parietal areas.

No group differences were identified in respect to the association between Verbal Fluency test performances and CVR CO_2_ maps. Figure 6 shows the Spearman’s rho maps referring to the relationship between the CVR CO_2_ maps and Shapes test. The SS and PP groups display a fairly similar pattern of correlation, showing a positive relationship between the CVR CO_2_ response and Shapes test performance in medial brain regions with negative rho values in frontal cortex. In contrast, the V group demonstrates an opposite pattern with negative medial correlations, along with positive associations represented in parietal areas. CVR CO_2_-Shapes test correlation was significantly higher in both the SS and PP groups compared to the V group in the right insular region and superior perisylvian areas (Figure 4B).

## Discussion

To our knowledge, this is the first study combining ASL perfusion and CVR CO_2_ measures with a longitudinal follow-up of cognitive abilities in elderly individuals with preserved neuropsychological performances at baseline. Our data indicate that both imaging measures show a subtle decrease in cases with the first signs of cognitive instability (V group) suggesting the presence of cerebral hypoperfusion and decreased cerebrovascular reactivity mainly in parietal and fronto-temporal association areas in this particular group. Importantly, cases with continuous cognitive decline (PP group) at high risk for MCI transition display an increase of both parameters in the same areas. The distinct profile of cognitively unstable cases compared to the two other groups of healthy controls is also documented by our clinicoradiologic correlations.

Early ASL-MRI contributions showed both hypoperfusion and hyperperfusion areas in MCI and AD cases stressing the brain efforts to compensate lesion invasion and cognitive loss. Despite controversial observations, two main patterns have been identified. A marked hypoperfusion in posterior cingulate cortex, precuneus and parietal cortex is already present in MCI cases (Johnson et al., 2005; Dai et al., 2009). In AD, a global decrease in blood flow is observed as compared to healthy controls, but region specific decrease in perfusion have also been detected (Austin et al., 2011). The hypoperfusion areas become more diffuse in clinically overt AD as to MCI including temporo-occipital and parieto-occipital cortices as well as orbitofrontal cortex (Alsop et al., 2000; Asllani et al., 2008; Fleisher et al., 2009; Austin et al., 2011; Bron et al., 2014; Haller et al., 2016). A hyperperfusion in hippocampus and basal ganglia was reported both in MCI and AD cases but also in non-symptomatic high risk APOE 𝜀4 carriers (Alsop et al., 2008; Dai et al., 2009; Fleisher et al., 2009; Ding et al., 2014). ASL data on cognitively preserved elderly persons are very scarce. They showed both hypoperfusion in frontal, parietal and cingulate areas but also a strong negative association between diffuse hyperperfusion in neocortical association areas and cognitive performances in APOE 𝜀4 carriers pointing to the presence of compensatory mechanisms explained by a pathological elevation of neural activity, inflammation or increased blood supply through vascular dilation or increased vascular density. In a longitudinal study, we first reported that decreased ASL values in posterior cingulate cortex were associated with subtle cognitive changes in cognitively intact elderly subjects (Xekardaki et al., 2015). More recently, ASL perfusion rates in medial frontal and anterior cingulate cortex predicted cognitive performances in a 4-year follow up of healthy elders (de Vis et al., 2018). Our longitudinal findings in carefully selected healthy controls shed some light into subtle changes of brain perfusion in the very initial stages of cognitive instability. In agreement with the observations made in MCI and AD cohorts, we found a subtle decrease of ASL values mainly in parietal cortex and precuneus (and to a lesser degree in temporal and frontal cortex) that is already present at inclusion in cases with cognitive fluctuations over a subsequent 3-year follow-up. PP cases showed increased ASL values in the same areas at inclusion (even higher than those in the SS group yet non-significant), but they deteriorate continuously suggesting that hyperperfusion in these areas is not an efficient defense against the neurodegenerative process. This idea is further supported by the CVR CO_2_ measures showing a slightly decreased brain cerebrovascular reactivity in V cases with a steady increase in PP cases mainly in parietal areas. As for ASL, early studies using BOLD functional MRI documented CVR CO_2_ decrease mainly in temporal, parietal and posterior cingulate areas in AD cases but also in hippocampus in MCI cases (Cantin et al., 2011; Yezhuvath et al., 2012; Richiardi et al., 2015). Taken together, these data imply the presence of an inverted U-shape pattern of regional blood flow and cerebral vasoreactivity in parietal cortex in old age that might predict subsequent cognitive fate. In fact, the cerebral vasoreactivity to CO_2_ slightly decreased in control cases with fluctuant cognitive performances, shows a compensatory increase in these with continuous decline prior to the MCI status before a marked decrease in MCI and AD cases. The clinico-radiologic correlations between Shapes test measures, an indicator of visual working memory, and both ASL and CVR CO_2_ data point further to the different behavior of V cases compared to both PP and SS cases. Similar differences were not observed in respect to Verbal fluency possibly because of the absence of significant associations between cognitive performance and imaging parameters for this test.

The biological significance of these observations remains matter of debate. Cortical hypometabolism is a core feature in preclinical AD and is associated with worst clinical evolution (Ewers et al., 2013; Besson et al., 2015; Mendes et al., 2018). However, increased glucose metabolism was also reported in amyloid-negative amnestic MCI cases and is thought to reflect compensatory mechanism to the neuronal damage occurring early in the disease process (for review see Ashraf et al., 2015). Whether the hypoperfusion observed mainly in parietal and posterior cingulate areas is causally related to AD or only its epiphenomenon remained unclear. Without bringing a definite answer, our data suggest that parietal and posterior cingulate cortex hypoperfusion is an early event in totally asymptomatic cases with fluctuations in cognitive performances within the normal range. It has been long thought that the hyperperfusion patterns in clinically overt AD and MCI may reflect increased neural activity as part of compensatory mechanisms aiming to counterbalance the cognitive decline. Alternatively, they may reflect alteration in brain vasculature due to increased angiogenesis or increased cerebrovascular reactivity possibly reflecting dysregulation of the neurovascular unit without any significant gain in terms of cognitive performance (for review see Zlatar et al., 2016; Sierra-Marcos, 2017). The present observations combining ASL and CVR CO_2_ measures clearly support the second hypothesis. In fact, our PP cases displayed higher ASL and CVR CO_2_ values in parietal areas prior to their cognitive decline. These results parallel the findings of Zlatar et al. (2016) in APOE 𝜀4 healthy carriers who reported a negative association between verbal memory function and ASL values in medial fronto-temporal and parietal cortex.

Strengths of the present study include the 3-year neuropsychological follow-up, careful exclusion of MCI and incipient AD cases, and combined use of ASL and CVR-CO_2_ techniques. Several limitations should, however, be considered when interpreting these data. In the absence of longer follow-up, the cognitive fate of PP and V cases remains uncertain. No CSF measures of tau and Aβ protein were available in this work so that the real extent of AD pathology remains unknown. Most importantly, the small sample size may mask subtle imaging differences between SS and PP as well as V cases. Future studies including PET amyloid and tau assessment of AD pathology as well as longer follow-up are warranted to define better the role of cerebrovascular mechanisms in the prediction of cognitive deterioration in asymptomatic elderly individuals.

## Author Contributions

All authors contributed to the concept and preparation of the manuscript. MvdT, SH, and PG were responsible for data interpretation. CR, SH, and PG collected the data.

## Conflict of Interest Statement

The authors declare that the research was conducted in the absence of any commercial or financial relationships that could be construed as a potential conflict of interest.

## Footnotes

1 http://fsl.fmrib.ox.ac.uk/fsl

2 http://fsl.fmrib.ox.ac.uk/fsl/fslwiki/BET

3 http://www.fmrib.ox.ac.uk/fslwiki/FLIRT

4 http://www.fmrib.ox.ac.uk/fslwiki/Fslutils

5 http://fsl.fmrib.ox.ac.uk/fsl/fslwiki/Randomize

6 https://fsl.fmrib.ox.ac.uk/fsl/fslwiki/GLM#Two_Groups_with_continuous_covariate_interaction

## References

[B1] Alsop D. C. Casement M. de Bazelaire C. Fong T. Press D. Z. (2008). Hippocampal hyperperfusion in Alzheimer’s disease. *NeuroImage* 42 1267–1274. 10.1016/j.neuroimage.2008.06.006 18602481 PMC2675915

[B2] Alsop D. C. Detre J. A. Grossman M. (2000). Assessment of cerebral blood flow in Alzheimer’s disease by spin-labeled magnetic resonance imaging. *Ann. Neurol.* 47 93–100. 10.1002/1531-8249(200001)47:1<93::AID-ANA15>3.0.CO;2-8 10632106

[B3] Ashraf A. Fan Z. Brooks D. Edison P. (2015). Cortical hypermetabolism in MCI subjects: a compensatory mechanism? *Eur. J. Nucl. Med. Mol. Imaging* 42 447–458. 10.1007/s00259-014-2919-z 25267349

[B4] Asllani I. Habeck C. Scarmeas N. Borogovac A. Brown T. R. Stern Y. (2008). Multivariate and univariate analysis of continuous arterial spin labeling perfusion MRI in Alzheimer’s disease. *J. Cereb. Blood Flow Metab.* 28 725–736. 10.1038/sj.jcbfm.9600570 17960142 PMC2711077

[B5] Austin B. P. Nair V. A. Meier T. B. Xu G. Rowley H. A. Carlsson C. M. (2011). Effects of hypoperfusion in Alzheimer’s disease. *J. Alzheimers Dis.* 26(Suppl. 3), 123–133. 10.3233/JAD-2011-0010 21971457 PMC3303148

[B6] Baddley A. Emslie H. Nimmo-Smith I. (1994). *Doors and People. A Test of Visual and Verbal Recall and Recognition.* Bury St Edmunds: Thames Valley Test Company.

[B7] Barberger-Gateau P. Commenges D. Gagnon M. Letenneur L. Sauvel C. Dartigues J. (1992). Instrumental activities of daily living as a screening tool for cognitive impairment and dementia in elderly community dwellers. *J. Am. Geriatr. Soc.* 40 1129–1134. 10.1111/j.1532-5415.1992.tb01802.x 1401698

[B8] Besson F. L. La Joie R. Doeuvre L. Gaubert M. Mézenge F. Egret S. (2015). Cognitive and brain profiles associated with current neuroimaging biomarkers of preclinical Alzheimer’s disease. *J. Neurosci.* 35 10402–10411. 10.1523/JNEUROSCI.0150-15.2015 26203136 PMC6605120

[B9] Bron E. E. Steketee R. M. Houston G. C. Oliver R. A. Achterberg H. C. Loog M. (2014). Diagnostic classification of arterial spin labeling and structural MRI in presenile early stage dementia. *Hum. Brain Mapp.* 35 4916–4931. 10.1002/hbm.22522 24700485 PMC6869162

[B10] Bruyer R. Tuyumbu B. (1980). Fluence verbale et lésions du cortex cerebral: performances et types d’erreurs. *L’Encéphale* 6 287–297. 7449726

[B11] Cantin S. Villien M. Moreaud O. Tropres I. Keignart S. Chipon E. (2011). Impaired cerebral vasoreactivity to CO2 in Alzheimer’s disease using BOLD fMRI. *NeuroImage* 58 579–587. 10.1016/j.neuroimage.2011.06.070 21745581

[B12] Chen Y. Wolk D. A. Reddin J. S. Korczykowski M. Martinez P. M. Musiek E. S. (2011). Voxel-level comparison of arterial spin-labeled perfusion MRI and FDG-PET in Alzheimer disease. *Neurology* 77 1977–1985. 10.1212/WNL.0b013e31823a0ef7 22094481 PMC3235355

[B13] Dai W. Lopez O. L. Carmichael O. T. Becker J. T. Kuller L. H. Gach H. M. (2009). Mild cognitive impairment and Alzheimer disease: patterns of altered cerebral blood flow at MR imaging. *Radiology* 250 856–866. 10.1148/radiol.2503080751 19164119 PMC2680168

[B14] de Vis J. B. Peng S. Chen X. Li Y. Liu P. Sur S. (2018). Arterial-spin-labeling (ASL) perfusion MRI predicts cognitive function in elderly individuals: a 4-year longitudinal study. *J. Magn. Reson. Imaging* 48 449–458. 10.1002/jmri.25833 29292540 PMC6028323

[B15] Ding B. Ling H.-W. Zhang Y. Huang J. Zhang H. Wang T. (2014). Pattern of cerebral hyperperfusion in Alzheimer’s disease and amnestic mild cognitive impairment using voxel-based analysis of 3D arterial spin-labeling imaging: initial experience. *Clin. Interv. Aging* 9 493–500. 10.2147/CIA.S58879 24707173 PMC3971940

[B16] Ewers M. Brendel M. Rizk-Jackson A. Rominger A. Bartenstein P. Schuff N. (2013). Reduced FDG-PET brain metabolism and executive function predict clinical progression in elderly healthy subjects. *Neuroimage Clin.* 4 45–52. 10.1016/j.nicl.2013.10.018 24286024 PMC3841292

[B17] Fällmar D. Haller S. Lilja J. Danfors T. Kilander L. Tolboom N. (2017). Arterial spin labeling-based Z-maps have high specificity and positive predictive value for neurodegenerative dementia compared to FDG-PET. *Eur. Radiol.* 27 4237–4246. 10.1007/s00330-017-4784-1 28374078 PMC5579184

[B18] Fleisher A. S. Podraza K. M. Bangen K. J. Taylor C. Sherzai A. Sidhar K. (2009). Cerebral perfusion and oxygenation differences in Alzheimer’s disease risk. *Neurobiol. Aging* 30 1737–1748. 10.1016/j.neurobiolaging.2008.01.012 18325636 PMC2746874

[B19] Folstein M. Folstein S. McHugh P. (1975). ”Mini-mental state”. A practical method for grading the cognitive state of patients for the clinician. *J. Psychiatr. Res.* 12 189–198. 10.1016/0022-3956(75)90026-6 1202204

[B20] Haller S. Montandon M. Rodriguez C. Ackermann M. Herrmann F. Giannakopoulos P. (2017). APOE^∗^E4 is associated with gray matter loss in the posterior cingulate cortex in healthy elderly controls subsequently developing subtle cognitive decline. *AJNR Am. J. Neuroradiol.* 38 1335–1342. 10.3174/ajnr.A5184 28495939 PMC7959915

[B21] Haller S. Zaharchuk G. Thomas D. L. Lövblad K. O. Barkhof F. Golay X. (2016). Arterial spin labeling perfusion of the brain: emerging clinical applications. *Radiology* 281 337–356. 10.1148/radiol.2016150789 27755938

[B22] Jenkinson M. Bannister P. Brady J. Smith S. (2002). Improved optimisation for the robust and accurate linear registration and motion correction of brain images. *Neuroimage* 17 825–841. 10.1006/nimg.2002.1132 12377157

[B23] Jenkinson M. Beckmann C. Behrens T. Woolrich M. Smith S. (2012). FSL. *NeuroImage* 67 782–860. 10.1016/j.neuroimage.2011.09.015 21979382

[B24] Johnson N. A. Jahng G.-H. Weiner M. W. Miller B. L. Chui H. C. Jagust W. J. (2005). Pattern of cerebral hypoperfusion in Alzheimer disease and mild cognitive impairment measured with arterial spin-labeling MR imaging: initial experience. *Radiology* 234 851–859. 10.1148/radiol.2343040197 15734937 PMC1851934

[B25] Kaplan E. F. Goodglass H. Weintraub S. (1983). *The Boston Naming Test.* Philadelphia, PA: Lea & Febiger.

[B26] Liu Y. Zhu X. Feinberg D. Guenther M. Gregori J. Weiner M. (2012). Arterial spin labeling MRI study of age and gender effects on brain perfusion hemodynamics. *Magn. Reson. Med.* 68 912–922. 10.1002/mrm.23286 22139957

[B27] Mendes A. Tezenas du Montcel S. Levy M. Bertrand A. Habert M. Bertin H. (2018). Multimorbidity is associated with preclinical Alzheimer’s disease neuroimaging biomarkers. *Dement. Geriatr. Cogn. Disord.* 45 272–281. 10.1159/000489007 29953971

[B28] Milner B. (1971). Interhemispheric differences in the localization of psychological processes in man. *Br. Med. Bull.* 27 272–277. 10.1093/oxfordjournals.bmb.a070866 4937273

[B29] Montagne A. Barnes S. R. Sweeney M. D. Halliday M. R. Sagare A. P. Zhao Z. (2015). Blood-brain barrier breakdown in the aging human hippocampus. *Neuron* 85 296–302. 10.1016/j.neuron.2014.12.032 25611508 PMC4350773

[B30] Mutch W. Mandell D. Fisher J. Mikulis D. Crawley A. Pucci O. (2012). Approaches to brain stress testing: BOLD magnetic resonance imaging with computer-controlled delivery of carbon dioxide. *PLoS One* 7:e47443. 10.1371/journal.pone.0047443 23139743 PMC3489910

[B31] Popa-Wagner A. Buga A. Popescu B. Muresanu D. (2015). Vascular cognitive impairment, dementia, aging and energy demand. A vicious cycle. *J. Neural Trans.* 122 47–54. 10.1007/s00702-013-1129-3 24337666

[B32] Reitan R. (1958). Validity of the Trail Making Test as an indicator of organic brain damage. *Percept. Mot. Skills* 8 271–296. 10.2466/pms.1958.8.3.271

[B33] Richiardi J. Monsch A. Haas T. Barkhof F. Van de Ville D. Radü E. (2015). Altered cerebrovascular reactivity velocity in mild cognitive impairment and Alzheimer’s disease. *Neurobiol. Aging* 16 33–41. 10.1016/j.neurobiolaging.2014.07.020 25146454

[B34] Riederer I. Bohn K. P. Preibisch C. Wiedemann E. Zimmer C. A. Förster S. (2018). Alzheimer disease and mild cognitive impairment: integrated pulsed arterial spin-labeling MRI and 18F-FDG PET. *Radiology* 288 198–206. 10.1148/radiol.2018170575 29762090

[B35] Schnider A. Hanlon R. E. Alexander D. N. Benson D. F. (1997). Ideomotor apraxia: behavioral dimensions and neuroanatomical basis. *Brain Lang.* 58 125–136. 10.1006/brln.1997.1770 9184099

[B36] Sierra-Marcos A. (2017). Regional cerebral blood flow in mild cognitive impairment and Alzheimer’s disease measured with arterial spin labeling magnetic resonance imaging. *Int. J. Alzheimers Dis.* 2017:5479597. 10.1155/2017/5479597 28573062 PMC5442339

[B37] Smith S. (2002). Fast robust automated brain extraction. *Hum. Brain Mapp.* 17 143–155. 10.1002/hbm.10062 12391568 PMC6871816

[B38] Smith S. Nichols T. (2009). Threshold-free cluster enhancement: addressing problems of smoothing, threshold dependence and localisation in cluster inference. *Neuroimage* 44 83–93. 10.1016/j.neuroimage.2008.03.061 18501637

[B39] Tosun D. Schuff N. Jagust W. Weiner M. W. (2016). Discriminative power of arterial spin labeling magnetic resonance imaging and 18F-fluorodeoxyglucose positron emission tomography changes for amyloid-β-positive subjects in the Alzheimer’s disease continuum. *Neurodegener. Dis.* 16 87–94. 10.1159/000439257 26560336

[B40] van der Thiel M. Rodriguez C. Giannakopoulos P. Burke M. X. Lebel M. Gninenko N. (2018). Brain perfusion measurements using multi-delay arterial spin labeling are systematically biased by number of delays. *Am. J. Neuroradiol.* 39 1432–1438. 29976831 10.3174/ajnr.A5717 PMC7410552

[B41] Wechsler D. A. (1955). *Manual for the Wechsler Adult Intelligence Scale.* New York, NY: Psychological Corporation.

[B42] Wechsler D. A. (1977). *Wechsler Memory Scale.* San Antonio, TX: Psychological Corporation.

[B43] Wierenga C. E. Hays C. C. Zlatar Z. Z. (2014). Cerebral blood flow measured by arterial spin labeling MRI as a preclinical marker of Alzheimer’s disease. *J. Alzheimers Dis.* 42 411–419. 10.3233/JAD-141467 25159672 PMC5279221

[B44] Xekardaki A. Rodriguez C. Montandon M. Toma S. Tombeur E. Herrmann F. (2015). Arterial spin labeling may contribute to the prediction of cognitive deterioration in healthy elderly individuals. *Radiology* 274 490–499. 10.1148/radiol.14140680 25291458

[B45] Yezhuvath U. S. Uh J. Cheng Y. Martin-Cook K. Weiner M. Diaz-Arrastia R. (2012). Forebrain-dominant deficit in cerebrovascular reactivity in Alzheimer’s disease. *Neurobiol. Aging* 33 75–82. 10.1016/j.neurobiolaging.2010.02.005 20359779 PMC2896562

[B46] Zhang Y. Brady M. Smith S. (2001). Segmentation of brain MR images through a hidden Markov random field model and the expectation-maximization algorithm. *IEEE Trans. Med. Imaging* 20 45–57. 10.1109/42.906424 11293691

[B47] Zigmond A. Snaith R. (1983). The hospital anxiety and depression scale. *Acta Psychiatr. Scand.* 67 361–370. 10.1111/j.1600-0447.1983.tb09716.x 6880820

[B48] Zlatar Z. Z. Bischoff-Grethe A. Hays C. C. Liu T. T. Meloy M. J. Rissman R. A. (2016). Higher brain perfusion may not support memory functions in cognitively normal carriers of the ApoE 𝜀4 allele compared to non-carriers. *Front. Aging Neurosci.* 8:151 10.3389/fnagi.2016.00151 PMC4919360 27445794

